# Fortified synbiotic dessert for improving malnutrition in hemodialysis patients: A randomized, double‐blind, controlled trial

**DOI:** 10.1002/fsn3.3728

**Published:** 2023-10-05

**Authors:** Farzaneh Azad, Maryam Hamidianshirazi, Seyed Mohammad Mazloomi, Maryam Shafiee, Maryam Ekramzadeh

**Affiliations:** ^1^ Student Research Committee, School of Nutrition and Food Sciences Shiraz University of Medical Sciences Shiraz Iran; ^2^ Department of Food Hygiene and Quality Control, Health and Food Quality Control, Food and Supplements Safety Research Center Shiraz University of Medical Sciences Shiraz Iran; ^3^ Shiraz Nephro‐Urology Research Center Shiraz University of Medical Sciences Shiraz Iran; ^4^ Department of Clinical Nutrition, Nutrition Research Center, School of Nutrition and Food Sciences Shiraz University of Medical Sciences Shiraz Iran

**Keywords:** calcium, functional food, hemodialysis, malnutrition, oxidative stress, prebiotic, probiotic, synbiotic, uremia, vitamin D

## Abstract

As dysbiosis of gut microbiota is recognized as a major risk factor for malnutrition in hemodialysis (HD) patients, we aimed to assess the effects of fortified synbiotic dessert on malnutrition, oxidative stress, inflammation, and quality of life in patients undergoing hemodialysis. A total of 50 hemodialysis patients were randomized into two groups of intervention and control to consume either 50 g of synbiotic dessert fortified with vitamin D (1000 IU) and calcium (500 mg) (FSD) or 50 g of control dessert (CD) for 8 weeks, respectively. Changes in nutritional status [Subjective Global Assessment (SGA)], anthropometric measures, malondialdehyde (MDA), total antioxidant capacity (TAC), high‐sensitivity C‐reactive protein (hs‐CRP), ferritin, biochemistry [serum albumin, vitamin D, creatinine, blood urea nitrogen (BUN), complete blood count (CBC), and electrolytes], and quality of life were assessed before and at the end of the trial. The SGA scores and serum ferritin levels decreased significantly in the FSD group compared to the control group (*p* = .01 and *p* = .03, respectively). Regarding other markers, no statistically significant changes were found comparing the two groups. This novel fortified synbiotic dessert as a functional food may be effective in reducing the severity of malnutrition by improving SGA score in short term in hemodialysis patients. Thus, it is suggested to do further studies to elucidate the possible mechanisms related to the effects of this dessert on microbiota, skeletal muscle mass, and inflammation in HD in long term.

## Graphical abstract

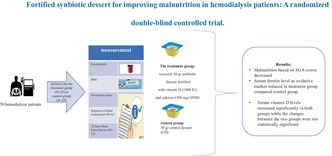

## 1 INTRODUCTION

Protein‐energy wasting (PEW) is a widespread consequence in patients under hemodialysis (Naini et al., 2016).

The link between malnutrition and inflammation is well established (Harvinder et al., 2015). Protein‐calorie malnutrition is secondary to inflammation. These two conditions are concurrent in hemodialysis (HD) patients which are known as malnutrition–inflammation complex syndrome (MICS) leading to poor dialysis outcomes such as cardiovascular disease, anemia, reduced quality of life, and higher mortality rate (Salehi et al., 2012). The potential triggers of MICS include oxidative stress (the most important one), uremia, nutrient loss throughout dialysis, a low nutrient intake due to anorexia, and comorbid diseases (Salehi et al., 2012). Intestinal dysbiosis is also another factor which can lead to the pathogenesis of systemic inflammation in HD patients (Shi et al., 2014). Recent evidence have asserted that the microbiome profile is altered in end‐stage renal disease (ESRD) patients undergoing hemodialysis (Duan et al., 2020). It is documented that the imbalance of gut microbiota can contribute to intestinal barrier disturbance and bacterial translocation, contributing to constant systemic inflammation in these patients (Xu et al., 2017). Dysbiosis also leads to harmful nephrovascular uremic toxin accumulation (Stanford et al., 2020), which in turn exacerbates dysbiosis. Therefore, the defective cycle of dysbiosis and uremic toxin accumulation continue to worsen (Mafra et al., 2019). Thus, with the aforementioned evidence, the correction of uremic toxin overload and dysbiosis is of great importance. Probiotics are defined as live microorganisms which can bring health benefits, if administered in adequate amounts (Lozupone et al., 2012). Probiotics have been offered as a promising adjuvant therapy in HD patients by improving inflammation, oxidative stress, and reducing uremic toxins (Vaziri et al., 2016). One of the most popular probiotics being widely used recently is *Bacillus coagulans*. This probiotic has a spore‐like protein layer that increases its survival and stability in the stomach and intestinal environment. This bacillus is known to be safe for long‐term use in humans (Armani et al., 2017). Prebiotics are known as nondigestible food compounds stimulating the growth and/or activity of probiotics (Koppe et al., 2015). Also, inulin as a common prebiotic has a role in improving antioxidant status and appetite (Koppe et al., 2015). The combination of prebiotics with probiotics (synbiotic) is proposed to have more health benefits in comparison with pre‐ or probiotics alone (Esmaeilinezhad et al., 2019).

In addition, excessive production of free radicals leads to the pathogenesis of chronic diseases. MDA is a useful indicator for estimating lipid peroxidation (Soleimani et al., 2017). The results of 13 clinical trials in CKD patients showed the beneficial effects of synbiotic compounds in improving oxidative stress and reducing malondialdehyde (MDA) levels (March et al., 2020). HD patients are also affected by vitamin D deficiency due to reduced kidney function leading to calcium deficiency which ultimately causes hyperparathyroidism and bone disorders. Thus, calcium and vitamin D supplementation is another important, effective, and routine intervention in HD patients (Thongprayoon et al., 2019). Also, vitamin D has been associated with preservation of muscle mass and lower inflammation in dialysis patients (Hashemi et al., 2013; Kelly, 2008). The levels of 1,25(OH)_2_D in the serum decrease gradually due to a deficiency in 25(OH)D together with the reduced ability of renal proximal tubular cells to utilize 25(OH)D, elevated levels of fibroblast growth factor (FGF)‐23, and decreased functioning of renal tissue (Mitchell et al., 2015). Vitamin D deficiency is linked to higher morbidity and all‐cause mortality rate and unfavorable outcomes (Mitchell et al., 2015; Stoyanova et al., 2011). Additionally, the continuous drop in serum calcitriol, resulting from vitamin D deficiency, leads to secondary hyperparathyroidism (SHPT) and its associated complications (Mitchell et al., 2015). The development of renal osteodystrophy is an outcome of disruptions in calcium and phosphorus metabolism caused by renal insufficiency (Gibson & Roberfroid, 1995). Decreased renal mass in HD patients leads to reduced synthesis of 1,25 (OH)_2_D and increased levels of plasma phosphorus which in turn causes a decrease in calcium absorption and lower levels of plasma calcium (Gibson & Roberfroid, 1995). Then, hypocalcemia results in higher parathyroid secretion and lower rate of bone calcification (Gibson & Roberfroid, 1995). This ultimately leads to the occurrence of osteitis fibrosa and osteomalacia, which are the primary constituents of renal osteodystrophy (Gibson & Roberfroid, 1995). Excessive vitamin D intake from food is challenging, and the body has built‐in mechanisms to control the production of vitamin D3 from sunlight exposure (Olveira & González‐Molero, 2016). Thus, taking vitamin D and calcium in the form of functional food would be a good option to neutralize the detrimental effects of vitamin D deficiency and hypocalcemia in HD patients.

Taking into account that malnutrition, inflammation, and oxidative stress are major characteristics of HD patients, providing these patients with a combination of synbiotics, vitamin D, and calcium, might alleviate the related comorbidities and mortality through improved nutritional status and oxidative balance.

Therefore, the aim of this study was to evaluate the efficacy of a synbiotic dessert fortified with calcium and vitamin D as a functional food on malnutrition, oxidative stress, and inflammation in the intervention group compared to the routine regimen in the control group in HD patients.

## 2 METHODS

### 2.1 Study design and patient characteristics

This 8‐week randomized, double‐blind, placebo‐controlled trial was conducted in ESRD patients who received regular hemodialysis. The study protocol was in accordance with the Declaration of Helsinki and Good Clinical Practice guidelines and approved by Ethics Committee (IR.SUMS.REC.1397.327). This study was registered in Iranian Registry of Controlled Trial (IRCT) (IRCT20100223003408N5) where the trial protocol could be obtained. Written informed consent was obtained from each eligible participant. CKD patients (stage 5) aged 20–80 years who received regular HD for at least 6 months twice a week (with polysulfone/polyamide membranes, reverse‐osmosis purified water, and bicarbonate‐containing dialysate), with any history of active infection, diabetes, cancer, autoimmune diseases, hospitalization, and any change in erythropoietin intake in the last month met eligible criteria. The exclusion criteria limited the study population by eliminating patients who were lactose intolerant, had serum levels of vitamin D above 50 ng/dL, and phosphorus above 5.5 mg/dL. In our survey, we considered the inclusion and exclusion criteria in patients' selection. Because of the limited number of eligible subjects in terms of serum phosphorus levels, this criterion was ignored. Since fortified dessert contained optimal phosphorus content and also patients received Renagel as a phosphate binder in cases of hyperphosphatemia, this elimination was acceptable.

All participants used Renagel as a phosphate binder and the dose was the same for both groups. A sample size of 17 patients per group was calculated based on MDA score (as primary outcome) with M1: 3.9, M2: 2.6, SD1: 2.6, and SD2: 0.3 at the end of the trial, and alpha error probability of 0.05, power = 0.80 and allocation ratio (N2/N1 = 1), according to a similar study (Jean et al., 2017). By evaluating dropout of 45%, we considered 25 patients in each group as the final sample size. A total of 280 HD patients were screened, among them 86 patients were eligible according to our inclusion and exclusion criteria and 50 of them signed the informed consent to participate in this study. The patients were randomly allocated to either the treatment group or the control group based on a computer‐generated chart with fixed‐size block of 4. Patients in treatment group received 50‐g fortified synbiotic dessert (FSD) per day and those in control group received 50 g nonfortified control dessert (CD) per day for 8 weeks. The content of both fortified and control dessert can be found in Table 1. In the process of preparing FSD after mixing the dry ingredients based on the predetermined amounts or percentages that were determined by various experimental procedures, the mixture was added to required amounts of 1.5% low‐fat milk. In the second stage, the mixture was heated at 65°C for 20 min in Ben Murray in order to be hydrated. Third stage was homogenization and pasteurization at 85°C for 20 min. Microbial and sensory analyses of the dessert (microbial count, appearance, flavor, and color) were done before beginning of the trial to ensure the safety and palatability of the product. The desserts were processed by Ramak Dairy Company. The amount of dessert in each package was 50 g that was found to be acceptable for patients. In the intervention group, 500‐mg calcium in fortified dessert was replaced by one of their 500‐mg calcium tablets as a routine regimen in HD patients, while in CD group patients continued consumption of calcium tablets. Other daily supplements, including vitamin D, were also used in both groups under supervision of nephrologist as routine. Participants consumed the desserts every day. Dessert packages were numbered for each patient based on the randomization sequence. Then, each patient received one type of dessert (FSD or CD) based on the given number. This trial was double blinded meaning that the patients and the main investigator were both unaware of the type of dessert. Desserts were delivered to patients by a colleague in a weekly dose. Patients were informed that they should keep the desserts in a refrigerator at 4°C. It is noteworthy that a clinical nutritionist recommended general dietary advice for CKD patients under hemodialysis in terms of protein, potassium, phosphorus, sodium, and fluid intake. The list of food substitutes was taught to all the patients by the nutritionist and they were asked to continue their routine diet during the study period. Also, routine hemodialysis care was monitored by a nephrologist for each patient over the 8 weeks.

**TABLE 1:** Fortified synbiotic and control dessert composition.^a^

Ingredients	Fortified synbiotic dessert	Placebo dessert
Energy (kcal)	38.74	40.39
Carbohydrate (gr)	6.43	6.43
Protein (gr)	1.89	2.2
Fat (gr)	0.61	0.66
Milk (1.5% fat)	81.53	87.745
Sugar (%)	6	6
Carrageenans (%)	0.35	0.35
Tapioca (modified starch) (%)	3.5	3.5
Gelatin (%)	1	1.4
Maltodextrin (%)	1	1
Vanilla (%)	0.005	0.005
Calcium citrate (%)	2.5	0
Vitamin D (%)	0.015	0
Inulin (%)	2	0
*Bacillus coagulans* bacterium (%)^b^	0.1	0
Dietary potassium content (mg)	30.7	33
Dietary phosphorus content (mg)	90.6	97.5

^a^ The amounts are calculated in 50 g of dessert.

^b^ Colony‐forming unit (2 × 10^11^ CFU/gr).

HD patients were observed at each session of hemodialysis by the main investigator to check their adherence, evaluate any potential adverse effects, and reply to any possible questions about dessert preservation or consumption. Also, they were requested to bring back the empty container of the dessert to be ensured about their consumption. The patients were also assessed by phone contact when they were at home on nondialysis days to monitor their compliance. Also, adverse events were examined by clinical staff in each dialysis session during the trial phase in all participants.

Primary outcomes of this study were absolute change in serum levels of MDA in patients. SGA is established as a comprehensive validated nutritional assessment tool and predictor of complications and clinical outcomes (Bauer et al., 2002; Soleimani et al., 2017). This questionnaire has several strengths, including: reliable, validate, inexpensive, no need to evaluate laboratory parameters, and convenient to use. Hence, during this study, the nutritional status of all patients was evaluated by SGA. This questionnaire was filled at baseline and end of the study through face‐to‐face interview and physical examinations by the main investigator. This questionnaire examined all changes including the weight change (during the last 6 months and 2 weeks), gastrointestinal symptoms that continued for more than 2 weeks, dietary intake, functional capacity, and physical examination (including muscle wasting, loss of subcutaneous fat, ankle edema, and ascites) (Bauer et al., 2002; Soleimani et al., 2017). Each of these factors was ranked as A, B, or C which show the severity of malnutrition. Then, these measures would be converted to numerical value. A score below 10 points is interpreted as well nourished; a score of 10–17 points is interpreted as at risk for malnutrition or mildly to moderately malnourished; and a score over 17 points is regarded as severely malnourished (Salehi et al., 2013). SF‐12 (short form 12) questionnaire was also filled out before and after the study. The validated Iranian version of SF‐12 questionnaire was applied in order to evaluate health‐related quality of life in patients under hemodialysis. It contains 12 questions in the context of 8 scales including general health, mental health, social function, physical function, role limitations due to physical health, emotional health, bodily pain, and vitality (Sohrabi et al., 2016). To evaluate dietary intake, 3‐day dietary recalls (including 2 weekdays and 1 weekend day) were obtained from patients at baseline and at the end of the trial. We analyzed dietary intakes by Nutritionist IV software (version 3.5.2; 1994, N‐Squared Computing, San Bruno, CA, USA). Weight and height were measured at the beginning and at the end of the study. Weight was measured with the least amount of cloth and without shoes with a digital scale with an accuracy of 0.1 kg and height was measured with standing position on the wall without shoes with an accuracy of 0.1 cm. Body mass index (BMI) was calculated using the formula (weight (kg)/height (m)^2^) (Lozupone et al., 2012). It should be noted that all the patients were in sedentary condition in terms of physical activity. Before and after the study, 5 cc blood samples were taken from each patient. The blood was taken from the patient's arm used for HD cannulae just before the beginning of the HD session. The serum was detached by centrifugation at 3000 ***g***/min for 5 min and stored at −70°C, until final measurement for serum levels of MDA, high‐sensitivity C‐reactive protein (hs‐CRP), ferritin, total antioxidant capacity (TAC), vitamin D, calcium, phosphorus, sodium, potassium, albumin, creatinine, blood urea nitrogen (BUN), and complete blood count (CBC). Serum concentrations of MDA were measured by the modified thiobarbituric acid method (spectrophotometric method) (Sohrabi et al., 2015). Serum levels of hs‐CRP were measured using highly sensitive ELISA kit (Monobind, Inc.) (Sohrabi et al., 2015). Serum ferritin levels were measured using ELISA kit (IBL, Germany) (Ekramzadeh et al., 2013). Serum TAC was determined by colorimetric assay using a specific assay kit (Biocore diagnostics, Hamburg, Germany) (Zal et al., 2007). The complete blood count (CBC) including hemoglobin, hematocrit, red blood cell (RBC) count, and white blood cell (WBC) count was measured electronically by an automated hematology analyzer (Esmaeilinezhad et al., 2020). Other biomarkers were evaluated with autoanalyzer.

### 2.2 Statistical analysis

The data were analyzed using the SPSS statistical software (v. 23, SPSS). Normal distribution of the data was checked by Kolmogorov–Smirnov test. Independent t test was used for between‐group analysis. Paired *t* test was used to assess changes in each group. *p* < .05 was considered as the significance level.

## 3 RESULTS

**FIGURE 1:**
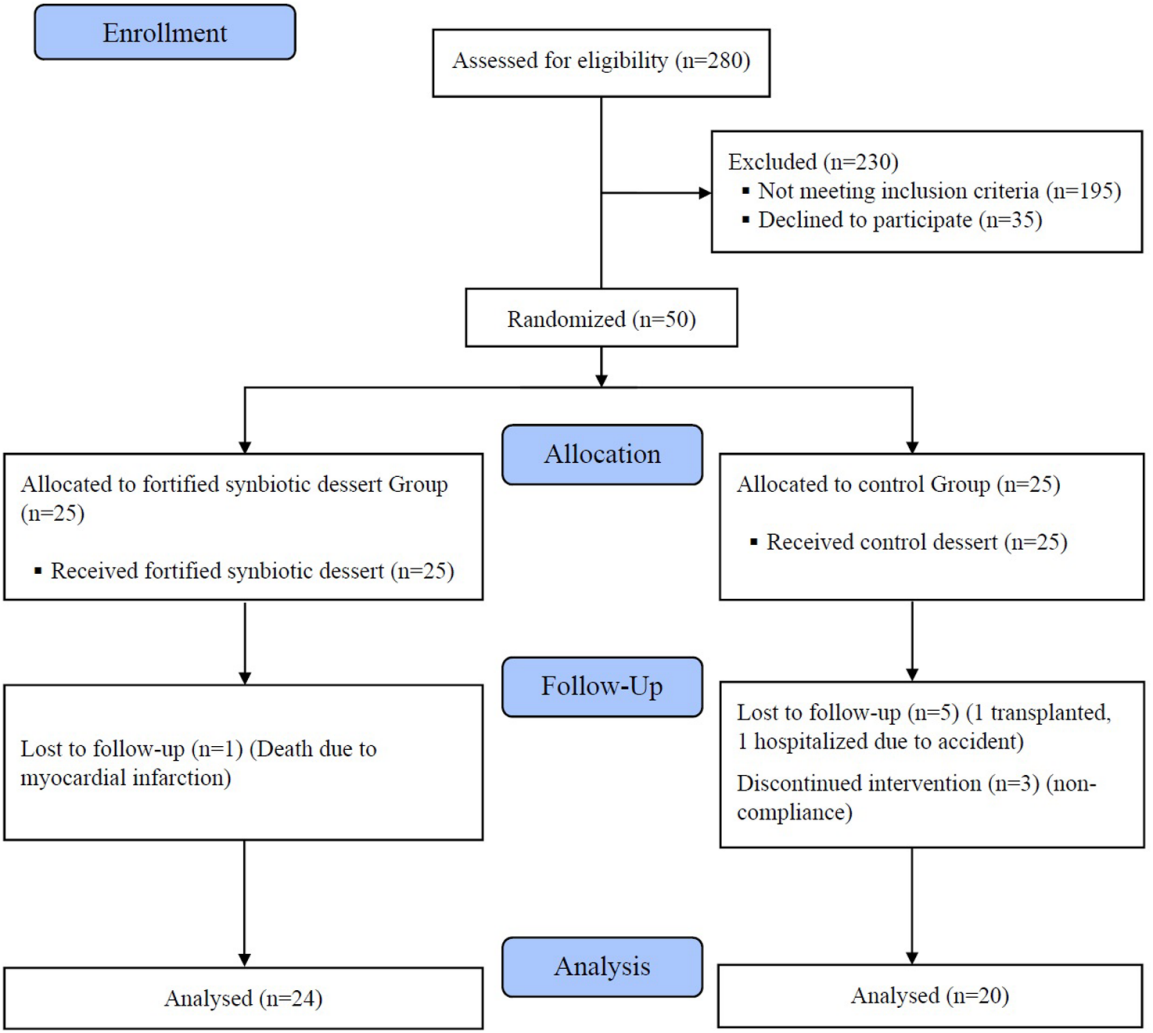
Flowchart of the trial.

The study was performed from July through November 2019. Seventy percent of patients in the control group and 45.8% of patients in the intervention group were male. As depicted in flow chart (Figure 1) during the study, one patient in FSD group and four in CD group left the study. Baseline demographic and laboratory parameters and also nutritional intake of study participants are shown in Table 2. No statistically significant differences were found between these two groups in terms of baseline characteristics. Changes in clinical outcomes during the treatment phase of the trial are demonstrated in Table 3. SGA scores decreased significantly in FSD group (*p* = .001), whereas reduction of SGA score in CD group was not statistically significant. When comparing the two groups, changes in these scores were statistically significant (*p* = .01) meaning that malnutrition based on SGA score decreased after consumption of FSD. Serum Vitamin D levels were not statistically significant (*p* = .58). Comparing the intervention and control group, ferritin changes were statistically significant (*p* = .03) which might be due to antioxidant properties of fortified symbiotic dessert. When comparing the two groups, other clinical variables and SF‐12 scores showed no statistically significant changes. Also, no intragroup and intergroup differences in food intake items were seen during the 8 weeks of the trial (Table 4).

**TABLE 2:** Baseline demographic characteristics and measured parameters in patients. ^a^ ^,^ ^b^

Demographic characteristics and measured parameters in patients	Fortified symbiotic dessert group (*n* = 24)	Control group (*n* = 20)	*p*‐Value
Age, y	52.33 ± 15.42	52.50 ± 15.27	.97
Males, *n* (%)	11 (45.8)	14 (70)	.10
Daily Kt/V	1.42 ± 0.26	1.34 ± 0.17	NS
Dialysis Duration (month)	62.75 ± 42.05	79.80 ± 59.71	.27
Weight (kg)	68.52 ± 18.37 [60.79 to 76.27]	67.8 ± 19.22 [58.80 to 76.79]	.90
BMI (kg/m^2^)	24.56 ± 3.96 [22.88 to 26.23]	24.02 ± 4.92 [21.71 to 26.32]	.68
SGA	13.70 ± 3.30 [12.31 to 15.10]	13.55 ± 2.81 [12.23 to 14.26]	.92
Albumin (gr/dL)	4.15 ± 0.39 [3.98 to 4.31]	4.12 ± 0.51 [3.88 to 4.36]	.80
BUN (mg/dL)	56.33 ± 13.91 [50.45 to 62.20]	54.75 ± 15.30 [47.58 to 61.91]	.72
Creatinine (mg/dL)	9.03 ± 2.15 [8.12 to 9.94]	8.67 ± 2.05 [7.71 to 9.63]	.58
Ferritin (ng/mL)	486.45 ± 228.25 [390.07 to 582.83]	413.90 ± 233.02 [304.83 to 522.96]	.30
MDA (μmol/L)	3.61 ± 0.89 [3.24 to 3.99]	3.50 ± 1.08 [2.99 to 4.01]	.48
TAC (mmol/L)	1.59 ± 0.23 [1.49 to 1.69]	1.59 ± 0.25 [1.47 to 1.71]	.95
hs‐CRP (ng/mL)	2820.54 ± 1182.43 [2321.24 to 3319.83]	2576.85 ± 1306.15 [1968.55 to 3188.14]	.52
Vitamin D (IU/mL)	25.61 ± 11.94 [20.56 to 30.65]	24.20 ± 8.71 [20.12 to 28.28]	.66
Calcium (mg/dL)	8.19 ± 0.58 [7.94 to 8.44]	8.28 ± 0.88 [7.86 to 8.69]	.70
Phosphorous (mg/dL)	4.86 ± 1.33 [4.30 to 5.42]	5.07 ± 1.20 [4.51 to 5.63]	.59
Sodium (mEq/L)	139.95 ± 2.98 [138.69 to 141.21]	139.45 ± 3.17 [137.96 to 140.93]	.46
Potassium (mEq/L)	5.42 ± 0.61 [5.15 to 5.68]	5.45 ± 0.97 [4.99 to 5.91]	.88
Hemoglobin (gr/dL)	10.88 ± 1.60 [10.21 to 11.56]	11.41 ± 1.92 [10.51 to 12.30]	.33
Hematocrit (%)	35.65 ± 4.96 [33.55 to 37.74]	36.17 ± 5 [33.83 to 38.51]	.73
RBC (10^6^/μL)	3.91 ± 0.51 [3.69 to 4.13]	3.94 ± 0.65 [3.63 to 4.25]	.86
WBC (/μL)	6262.50 ± 1995.82 [5419.73 to 7105.26]	6330 ± 1160.35 [5786.93 to 6873.06	.89
PHS	0.61 ± 0.20 [0.53 to 0.70]	0.55 ± 0.18 [0.47 to 0.64]	.31
MHS	0.67 ± 0.22 [0.57 to 0.77]	0.69 ± 0.21 [0.59 to 0.79]	.78
TS	0.65 ± 0.18 [0.57 to 0.72]	0.62 ± 0.19 [0.52 to 0.71]	.60
Energy (kcal/day)	1380.81 ± 669.17 [1098.24 to 1663.38]	1561.05 ± 478.39 [1337.15 to 1784.95	.31
Dietary carbohydrate (gr/day)	170.74 ± 103.89 [126.87 to 214.61]	188.51 ± 76.47 [152.72 to 224.30]	.20
Dietary protein (gr/day)	46.31 ± 31.42 [33.04 to 59.58]	53.13 ± 19.31 [44.09 to 62.16]	.07
Dietary fat (gr/day)	57.52 ± 22.75 [47.91 to 67.13]	66.91 ± 25.15 [55.14 to 78.68]	.20
Dietary calcium (mg)	260.26 ± 192.61 [178.92 to 341.59]	252.08 ± 109.91 [200.63 to 303.52]	.55
Dietary phosphorous (mg)	424.10 ± 258.82 [314.80 to 533.39]	497.37 ± 206.57 [400.68 to 594.05]	.31
Dietary sodium (mg)	2321.54 ± 839.20 [1967.17 to 2675.90]	2581.25 ± 703.20 [2252.13 to 2910.36]	.23
Dietary potassium (mg)	781.14 ± 520.34 [561.41 to 1000.86]	1027.59 ± 657.48 [719.87 to 1335.30]	.05
Dietary vitamin D (μg)	0.31 ± 1.06 [−0.13 to 0.76]	0.31 ± 0.81 [−0.06 to 0.69]	.95

Abbreviations: BMI, body mass index; BUN, blood urea nitrogen; hs‐CRP, high‐sensitivity C‐reactive protein; MDA, malondialdehyde; MHS, mental health score; PHS, physical health score; RBC, red blood cells; SGA, subjective global assessment; TAC, total antioxidant capacity; TS, Quality of life (total score); WBC, white blood cells.

^a^ Data are expressed as means ± SDs [95% confidence interval] or *n* (%).

^b^ Qualitative variables were examined with Chi‐square; quantitative variables were tested with independent *t*‐test.

**TABLE 3:** Effect of intervention on levels of measured parameters in patients.^a^

Measured parameters	Fortified symbiotic dessert group (*n* = 24)			Control group (*n* = 20)			*p*‐Value^c^
	Before	After	Change	Before	After	Change	
Weight (kg)	68.52 ± 18.37 [60.79 to 76.27]	68.22 ± 17.39 [60.88 to 75.57]	−0.29 ± 2.30 [−1.26 to 0.68]	67.8 ± 19.22 [58.80 to 76.79]	67.78 ± 19.06 [58.86 to 76.70]	−0.01 ± 2.29 [−1.09 to 1.06]	.69
BMI (kg/m^2^)	24.56 ± 3.96 [22.88 to 26.23]	24.48 ± 3.66 [22.93 to 26.03]	−0.07 ± 0.83 [−0.43 to 0.27]	24.02 ± 4.92 [21.71 to 26.32]	24.05 ± 4.92 [21.74 to 26.35]	0.03 ± 0.80 [−0.34 to 0.40]	.67
SGA	13.70 ± 3.30 [12.31 to 15.10]	12.66 ± 2.66 [11.51 to 14.06]	−1.04 ± 1.23^b^ [−1.45 to −0.37]	13.55 ± 2.81 [12.23 to 14.26]	13.40 ± 2.90 [12.08 to 14.41]	−0.15 ± 1.08 [−0.67 to 0.07]	.01^c^
Albumin (gr/dL)	4.15 ± 0.39 [3.98 to 4.31]	4.10 ± 0.42 [3.92 to 4.28]	−0.04 ± 0.25 [−0.15 to 0.06]	4.12 ± 0.51 [3.88 to 4.36]	4.15 ± 0.40 [3.96 to 4.34]	0.03 ± 0.32 [−0.12 to 0.18]	.21
BUN (mg/dL)	56.33 ± 13.91 [50.45 to 62.20]	50.45 ± 10.85 [45.87 to 55.04]	−5.87 ± 12.23^b^ [−28.56 to −16.85]	54.75 ± 15.30 [47.58 to 61.91]	50.40 ± 13.79 [43.94 to 56.85]	−4.35 ± 18.36 [−26.50 to −11.42]	.74
Creatinine (mg/dL)	9.03 ± 2.15 [8.12 to 9.94]	8.78 ± 1.94 [7.96 to 9.60]	−0.25 ± 1.53 [−0.89 to 0.39]	8.67 ± 2.05 [7.71 to 9.63]	9.03 ± 2.26 [7.96 to 10.09]	0.35 ± 1.41 [−0.31 to 1.01]	.18
Ferritin (ng/mL)	486.45 ± 228.25 [390.07 to 582.84]	390.45 ± 233.38 [291.91 to 489]	−96 ± 314 [−229 to 37]	413.90 ± 233.02 [304.83 to 522.96]	537.35 ± 365.93 [366.08 to 708.61]	123.45 ± 356.78 [−43.53 to 290.43]	.03^c^
MDA (μmol/L)	3.61 ± 0.89 [3.24 to 3.99]	3.58 ± 0.91 [3.19 to 3.96]	−0.03 ± 0.92 [−0.42 to 0.35]	3.50 ± 1.08 [2.99 to 4.01]	3.53 ± 1.09 [3.02 to 4.05]	0.03 ± 1.23 [−0.54 to 0.61]	.26
TAC (mmol/L)	1.59 ± 0.23 [1.49 to 1.69]	1.55 ± 0.24 [1.44 to 1.65]	−0.04 ± 0.20 [−0.13 to 0.04]	1.59 ± 0.25 [1.47 to 1.71]	1.64 ± 0.32 [1.48 to 1.79]	0.05 ± 0.36 [−0.11 to 0.22]	.27
hs‐CRP (ng/mL)	2820.54 ± 1182.43 [2321.24 to 3319.83]	2774.70 ± 1265.72 [2240.24 to 3309.17]	−45.83 ± 1537.49 [−695.06 to 603.39]	2576.85 ± 1306.15 [1965.55 to 3188.14]	2605.10 ± 1568.67 [1870.93 to 3339.26]	28.25 ± 1407.68 [−630.56 to 687.06]	.86
Vitamin D (IU/mL)	25.61 ± 11.94 [20.56 to 30.65]	30.10 ± 13.14 [24.55 to 35.65]	4.49 ± 5.65^b^ [2.10 to 6.88]	24.20 ± 8.71 [20.12 to 28.28]	28.13 ± 10.08 [23.41 to 32.85]	3.92 ± 8.27^b^ [0.05 to 7.79]	.78
Calcium (mg/dL)	8.19 ± 0.58 [7.94 to 8.44]	8.57 ± 0.79 [8.23 to 8.91]	0.37 ± 1.03 [−0.05 to 0.81]	8.28 ± 0.88 [7.86 to 8.69]	8.36 ± 0.66 [8.05 to 8.67]	0.08 ± 0.73 [−0.26 to 0.43]	.29
Phosphorous (mg/dL)	4.86 ± 1.33 [4.30 to 5.42]	4.88 ± 1.38 [4.30 to 5.47]	0.02 ± 1.47 [−0.60 to 0.64]	5.07 ± 1.20 [4.51 to 5.63]	4.83 ± 1.01 [4.36 to 5.30]	−0.24 ± 1.29 [−0.84 to 0.36]	.33
Sodium (mEq/L)	139.95 ± 2.98 [138.69 to 141.21]	139.66 ± 2.82 [138.47 to 140.85]	−0.29 ± 3.68 [−1.84 to 1.26]	139.45 ± 3.17 [137.96 to 140.93]	138.65 ± 2.25 [137.59 to 139.70]	−0.80 ± 3.20 [−2.3 to 0.70]	.72
Potassium (mEq/L)	5.42 ± 0.61 [5.15 to 5.68]	5.40 ± 0.74 [5.08 to 5.71]	−0.02 ± 0.12 [−0.27 to 0.23]	5.45 ± 0.97 [4.99 to 5.91]	5.64 ± 0.80 [5.26 to 6.02]	0.19 ± 1.12 [−0.33 to 0.71]	.43
Hemoglobin (gr/dL)	10.88 ± 1.60 [10.21 to 11.56]	10.36 ± 1.73 [9.62 to 11.09]	−0.52 ± 1.20^b^ [−1.03 to −0.01]	11.41 ± 1.92 [10.51 to 12.30]	11.17 ± 1.45 [10.49 to 11.84]	−0.24 ± 2.07 [−1.21 to 0.73]	.57
Hematocrit (%)	35.65 ± 4.96 [33.55 to 37.74]	33.62 ± 4.61 [31.67 to 35.56]	−2.02 ± 3.86^b^ [−3.66 to −0.39]	36.17 ± 5 [33.83 to 38.51]	35.78 ± 4.72 [33.57 to 37.99]	−0.39 ± 4.43 [−2.46 to 1.68]	.19
RBC (10^6^/μL)	3.91 ± 0.51 [3.69 to 4.13]	3.87 ± 0.59 [3.62 to 4.12]	−0.04 ± 0.47 [−0.24 to 0.16]	3.94 ± 0.65 [3.63 to 4.25]	4.14 ± 0.71 [3.80 to 4.47]	0.19 ± 0.56 [−0.06 to 0.46]	.14
WBC (/μL)	6262.50 ± 1995.82 [5419.73 to 7105.26]	6016.66 ± 1953.29 [5191.86 to 6841.47]	−245.83 ± 1434.65 [−851.63 to 359.96]	6330 ± 1160.35 [5786.93 to 6873.06]	6415 ± 1416.91 [5751.86 to 7078.13]	85 ± 949.39 [−359.32 to 529.32]	.38
PHS	0.61 ± 0.20 [0.53 to 0.70]	0.61 ± 0.24 [0.51 to 0.71]	−0.006 ± 0.11 [−0.06 to 0.57]	0.55 ± 0.18 [0.47 to 0.64]	0.51 ± 0.22 [0.51 to 0.71]	−0.04 ± 0.09^b^ [−0.09 to −0.002]	.33
MHS	0.67 ± 0.22 [0.57 to 0.77]	0.67 ± 0.26 [0.55 to 0.78]	−0.003 ± 0.11 [−0.05 to 0.04]	0.69 ± 0.21 [0.59 to 0.79]	0.69 ± 0.22 [0.59 to 0.80]	0.005 ± .078 [−0.03 to 0.04]	.78
TS	0.65 ± 0.18 [0.57 to 0.72]	0.64 ± 0.22 [0.55 to 0.74]	−0.006 ± 0.11 [−0.05 to 0.04]	0.62 ± 0.19 [0.52 to 0.71]	0.60 ± 0.21 [0.50 to 0.70]	−0.01 ± 0.05 [−0.04 to 0.008]	.72

Abbreviations: BMI, body mass index; BUN, blood urea nitrogen; hs‐CRP, high‐sensitivity C‐reactive protein; MDA, malondialdehyde; MHS, mental health score; PHS, physical health score; RBC, red blood cells; SGA, subjective global assessment; TAC, total antioxidant capacity; TS, Quality of life (total score); WBC, white blood cells.

^a^ Data are expressed as means ± SDs [95% confidence interval].

^b^ Significant *p*‐value within group.

^c^ Significant *p*‐value between groups.

**TABLE 4:** Dietary intakes of the participants during the intervention.^a^

	Fortified symbiotic dessert group (*n* = 24)	Control group (*n* = 20)	*p*‐Value^b^
Energy (kcal/day)	33.70 ± 172.02 [−38.93 to 106.34]	14.99 ± 149.52 [−54.98 to 84.97]	.70
Carbohydrate (gr/day)	3.61 ± 29.91 [−9.02 to 16.24]	6.45 ± 22.80 [−4.22 to 17.12]	.86
Protein (gr/day)	−0.70 ± 9.32 [−4.64 to 3.22]	−1.16 ± 6.94 [−4.41 to 2.08]	.23
Fat (gr/day)	0.24 ± 1.90 [−0.75 to 6.17]	−0.68 ± 10.85 [−5.75 to 4.39]	.24
SFA (gr/day)	2.71 ± 8.21 [−0.87 to 0.89]	−0.28 ± 1.49 [−0.98 to 0.41]	.53
MUFA (gr/day)	0.74 ± 2.93 [−0.49 to 1.98]	−0.51 ± 2.52 [−1.70 to 0.66]	.71
PUFA (gr/day)	0.24 ± 1.90 [−0.55 to 1.05]	0.03 ± 4.88 [−2.25 to 2.31]	.84
Fiber (gr/day)	0.47 ± 2.97 [−0.77 to 1.73]	0.05 ± 0.33 [−0.10 to 0.20]	.40
Cholesterol (mg)	−4.47 ± 39.70 [−21.24 to 12.28]	−19.03 ± 74.41 [16.63 to −0.53]	.66
Calcium (mg)	7.40 ± 59.97 [−17.92 to 32.72]	34.98 ± 161.36 [−40.53 to 110.50]	.57
Phosphorous (mg)	−4.47 ± 39.70 [−9.71 to 124.07]	9.03 ± 106.01 [−40.58 to 58.64]	.20
Sodium (mg)	68.62 ± 557.32 [−166.71 to 303.96]	−16.15 ± 386.54 [−197.06 to 164.76]	.80
Potassium (mg)	68.49 ± 307.17 [−61.21 to 198.20]	43.70 ± 200 [−50.01 to 137.42]	.86
Iron (mg)	0.25 ± 1.48 [−0.36 to 0.88]	0.001 ± 0.44 [−0.20 to 0.21]	.11
Vitamin B6 (mg)	0.13 ± 0.48 [−0.06 to 0.33]	−0.02 ± 0.14 [−0.09 to 0.04]	.08
Vitamin B9 (μg)	9.08 ± 26.26 [−2 to 20.17]	−2.79 ± 38 [−20.58 to 14.99]	.22
Vitamin B12 (μg)	−0.01 ± 0.17 [−0.08 to 0.06]	−0.03 ± 0.62 [−0.33 to 0.25]	.71
Vitamin D (μg)	−0.02 ± 0.13 [−0.08 to 0.02]	0.25 ± 1.14 [−0.27 to 0.78]	.16

Abbreviations: MUFA, monounsaturated fatty acids; PUFA, polyunsaturated fatty acids; SFA, saturated fatty acid.

^a^ Data are expressed as differences between baseline and after 8 weeks' intervention values (means ± SDs [95% confidence interval]).

^b^ Significant *p*‐value between groups.

## 4 DISCUSSION

To the best of our knowledge, this study is the first to examine the effect of FSD with calcium and vitamin D on malnutrition and inflammation indices in HD patients. In this randomized, double‐blind, controlled trial, FSD improved malnutrition in HD patients.

Our data indicated that malnutrition status evaluated by SGA score significantly improved in the FSD group compared to the control group. Although the difference in SGA was quite low between the two intervention and control groups, it was statistically significant. As we mentioned earlier, this synbiotic‐fortified dessert with *Bacillus coagulans*, inulin, vitamin D, and calcium was firstly examined. Oxidative stress and inflammation as a common consequence in HD patients leads to malnutrition, atherosclerosis, and other cardiovascular diseases (Jean et al., 2017; Vis & Huisman, 2016). Malnutrition is established as a prevalent complication in HD patients and is recognized as a pivotal risk factor for cardiovascular mortality (Ekramzadeh et al., 2013; Spatola et al., 2018). Therefore, the correction of malnutrition in HD patients is of great value. A missing link to explain the improvement of malnutrition based on SGA after consumption of symbiotic dessert in HD patients could be the mutual association between serum concentrations of low dialytic clearance uremic toxins [p‐cresyl sulfate (p‐CS) and indoxyl sulfate (IS)] and composition of gut microbiota. After initiation of dialysis therapy intermediate‐weight molecules including uremic toxins, peptides, and cytokines are only partially cleared. So, uremic accumulation still remains a great challenge in HD patients. Thus, uremic anorexia as a result of uremic intoxication and renal retention of middle‐sized molecules (MMs) contributes to malnutrition through decreased intake of protein, energy, and other macronutrients. The inhibitory effect of uremic toxins such as MMs on ingestive behavior in HD patients seems to be related to brain‐derived control of appetite regarding leptin and Melanocortin 4 receptor (MC4_R) (Carrero et al., 2008). Serum levels of PCS and IS are strong predictors of uremic syndrome, cardiovascular events, and mortality in HD population. Generation of these toxins may be associated with gut microflora by protein fermentation. Thus, targeted efforts to reduce PCS and IS through improved blood clearance are of great value (Meijers et al., 2009). In addition, in uremic patients, the combination and function of gut microflora are disrupted due to abnormal intestinal movements. It could lead to malnutrition, local and systemic inflammation, and other complications (Dehghani et al., 2016; Pan et al., 2020). Consumption of synbiotic meal may reduce ammonium hydroxide production from ammonia which subsequently acidifies colonic environment and causes a decrease in fecal PH and an increase in short‐chain fatty acids in colon in HD subjects. As these nitrogenous toxins are excreted mainly via feces, the urea concentration and ammonia production rate are correlated well with fecal PH. This acidification also modifies the intestinal microbiota (increasing the number of anaerobic bacteria and decreasing proteolytic bacteria) in a way that lowers intestinal permeability. Accordingly, diffusion of uremic wastes from the intestinal lumen to blood flow would be decreased (Lopes et al., 2019). Furthermore, presence of probiotics in gastrointestinal tract leads to better food digestion and absorption of certain nutrients and vitamins, accordingly enhances food intake and malnutrition (Dehghani et al., 2016). In line with our results, Soleimani et al. (Jean et al., 2017) showed that 12 weeks of intervention with probiotic supplementation in HD patients can significantly reduce SGA and improve malnutrition. Supplementation with probiotics has also been shown to improve malnutrition in other diseases such as inflammatory bowel syndrome and human immunodeficiency virus (HIV) (Kerac et al., 2009; Oh et al., 2019). The results of a study by Oh JH et al indicated that probiotic supplementation in patients with inflammatory bowel syndrome can improve malnutrition through a decline in SGA score (Oh et al., 2019). However, in Kerac et al. study, synbiotic supplementation failed to improve malnutrition in malnourished children with HIV (Kerac et al., 2009) Regarding the association of vitamin D and malnutrition, it has been shown that protein‐energy malnutrition is associated with vitamin D deficiency in HD patients (Fiedler et al., 2011). Binding of vitamin D to vitamin D receptor (VDR) on muscle fibers increases their size and improves muscle strength and physical function (Remelli et al., 2019). Furthermore, since inflammation and oxidative stress are considered as the main contributing factors in malnutrition, vitamin D can attenuate malnutrition–inflammation complex which is common in HD patients through inducing anti‐inflammatory effect (Ghone et al., 2013; Hansen et al., 2014). Thus, malnutrition improvement in this study might be the result of synergistic effects of both synbiotic and vitamin D consumed through the dessert in 8 weeks. In our study, after 8 weeks of intervention, serum ferritin levels had a slight decrease in FSD group and a slight increase in the CD group, none of them was statistically significant. But in comparison between groups, the changes were statistically significant. This result could be reflective of suppressing oxidative stress in the intervention group while this trend was accelerated in the control group. Ferritin is considered as a cellular antioxidant to control ROS production by suppressing free radical production. Thus, in cases of inflammatory conditions such as CKD and HD patients, ferritin does not reflect iron storage. It could be a surrogate marker of oxidative stress in HD patients as evidence reported that serum ferritin levels increase 2‐ to 3‐fold in infection and inflammatory processes. Although probiotics have been shown to increase iron absorption, we cannot predict an increase in iron absorption from the ferritin level due to inflammation in hemodialysis (Kalantar‐Zadeh et al., 2004) and ESRD patients (Jairam et al., 2010). Based on previous evidence, synbiotics and vitamin D could reduce oxidative stress and inflammation (Kooshki et al., 2019; Remelli et al., 2019) in HD patients which is consistent with our findings regarding ferritin. The results of current study did not show any statistically significant differences between the two groups regarding the oxidative stress (MDA and TAC) and inflammatory (hs‐CRP) markers. Contrary to our results, various studies have shown the positive effect of probiotics on improving inflammation and oxidative stress in patients under hemodialysis (Jean et al., 2017; Pan et al., 2020; Thongprayoon et al., 2019; Wang et al., 2015). The lack of change in inflammatory and oxidative stress markers in our study could be due to the short duration of intervention, different types of probiotics, and lower number of patients. Serum vitamin D was significantly increased in both FSD and CD groups after 8 weeks compared to the beginning of the study; however, this increment was higher in intervention group. No statistically significant difference was found between groups. Maybe if higher doses of vitamin D had been consumed through FSD for longer duration, significant change would have been observed comparing the two groups. This increment in serum vitamin D level in FSD group which was greater than control group could have some clinical value. It might be indicative of practical synergistic effects of fortified food intake, calcium, and vitamin D supplementation in patients consuming FSD. In ESRD patients, the kidneys are unable to produce active vitamin D, resulting in calcium deficiency. To compensate for calcium deficiency, PTH secretion increases. On the other hand, elevated serum phosphorus concentration within 4–8 h after dialysis (Agar et al., 2011) increases the secretion of PTH and fibroblast‐23 growth factor‐α which both inhibits renal alpha 1‐hydroxylase activity. As a result, it further reduces vitamin D levels, followed by lower calcium levels and eventually secondary hyperparathyroidism (Palmquist, 2006; Ranganathan et al., 2005). Therefore, calcium and vitamin D supplementation is one of the most important and routine pharmacological interventions in HD patients. No intragroup and intergroup differences for serum electrolytes (calcium, phosphorus, sodium, and potassium) were observed. In the present study, the control group continued to take calcium tablets while in the intervention group, calcium supplementation was discontinued and replaced by the synbiotic dessert fortified with calcium and vitamin D. Therefore, based on our results if calcium can be provided to HD patients in the form of calcium‐fortified foods instead of taking calcium tablets, the effects would be the same. Since HD patients take a wide range of pills and supplements to improve their symptoms, reducing the number of daily pills and capsules could be a good alternative in this population. We did not observe any significant change in serum albumin levels after the intervention comparing the two groups. Regarding CBC, no statistically significant difference was found between the two groups.

The strength point of this research was that strain of coagulans as probiotic for the first time in HD patients was used in dessert fortification which has more bioavailability and does not change during the pasteurization process. The observed effects related well to the synergistic effects of synbiotic, vitamin D, and calcium. If each dessert ingredient had been tested separately, different results might have been found. Ideal at least a third group without calcium and vitamin D, to assess the symbiotic effect. Also, it is noteworthy to say that the generalizability of current findings was reinforced by the multicenter design of the trial as the HD patients were selected from three major hemodialysis centers. Low sample size and short duration are some of the limitations of our study. Eight weeks is too short to observe the changes in primary and secondary outcomes, especially in terms of inflammatory and oxidative stress markers. It would be better to check other inflammatory factors such as interloukin‐6 (IL‐6), tumor necrosis factor‐alpha (TNF‐α), and interferon gamma (IFNγ) along with C‐reactive protein to make more sure of the effect of the intervention on inflammation. We also suggest assessing total iron‐binding capacity (TIBC), serum iron, malnutrition–inflammation score (MIS), body composition parameters, blood pressure, fecal microbiota test, and residual kidney function in future studies.

## 5 CONCLUSION

In summary, this study showed that pro‐ and prebiotic fortified with vitamin D and calcium supplementation in the form of a new functional food which was a synbiotic dessert in the intervention group compared to drug supplementation in the control group improved malnutrition within a short space of time in terms of SGA score in HD patients. This effect might be due to synergistic antioxidant and anti‐inflammatory properties of the pre/probiotics and vitamin D. Thus, an FSD as nutraceuticals might be regarded as a beneficial complementary regimen in treatment of malnutrition in HD patients.

## AUTHOR CONTRIBUTIONS

**Farzaneh Azad:** Data curation (equal); formal analysis (equal); investigation (equal); methodology (equal); project administration (equal); software (equal); writing – original draft (lead). **Maryam Hamidianshirazi:** Conceptualization (equal); data curation (equal); formal analysis (equal); investigation (equal); methodology (equal); project administration (equal); software (equal); visualization (equal); writing – review and editing (equal). **Seyed Mohammad Mazloomi:** Conceptualization (supporting); data curation (equal); funding acquisition (supporting); investigation (supporting); methodology (equal); project administration (supporting); resources (equal); supervision (supporting); validation (supporting); visualization (equal); writing – review and editing (equal). **Maryam Shafiee:** Conceptualization (supporting); investigation (supporting); methodology (equal); project administration (supporting); resources (equal); supervision (supporting); validation (supporting); visualization (supporting); writing – review and editing (equal). **Maryam Ekramzadeh:** Conceptualization (lead); data curation (equal); formal analysis (equal); funding acquisition (lead); investigation (equal); methodology (lead); project administration (lead); resources (equal); supervision (lead); validation (equal); visualization (lead); writing – original draft (supporting); writing – review and editing (lead).

## FUNDING INFORMATION

This study was extracted from an MSc thesis and financially supported by the research vice chancellor of (Shiraz University of Medical Sciences) SUMS (Grant No. 96–01–84‐16479).

## CONFLICT OF INTEREST STATEMENT

The authors declare that they have no relevant conflict of interest. The results presented in this paper have not been published previously in whole or part.

## ETHICS STATEMENT

This study was approved by the Ethics Committee of Shiraz University of Medical Sciences (IR.SUMS.REC.1397.327). All methods in the present study were performed in accordance with the principles of the relevant guidelines and regulations of Declaration of Helsinki, which is a statement of ethical principles that directs physicians and other participants in medical research involving human subjects.

## CONSENT

Informed consent was also obtained from the patients involved in the study after explaining the aim, method, and goal of the research.

## Data Availability

The data that support the findings of this study are available on request from the corresponding author.
